# Clinical-functional characteristics of children with asthma and obstructive sleep apnea overlap associated with attention deficit hyperactivity disorder: A cross-sectional study

**DOI:** 10.3389/fneur.2022.1097202

**Published:** 2023-01-09

**Authors:** Le Nguyen-Ngoc-Quynh, Mai Nguyen-Thi-Thanh, Mai Nguyen-Thi-Phuong, Chi Le-Quynh, Huong Le-Thi-Minh, Sy Duong-Quy

**Affiliations:** ^1^Department of Allergy, Immunology, and Rheumatology, National Children's Hospital, Hanoi, Vietnam; ^2^Department of Pediatrics, Hanoi Medical University, Hanoi, Vietnam; ^3^Pediatric Centre, Vinmec Times City International Hospital, Hanoi, Vietnam; ^4^Sleep Lab Center, Lam Dong Medical College and Bio-Medical Research Center, Dalat, Vietnam; ^5^Immuno-Allergology Division, Hershey Medical Center, Penn State Medical College, Hershey, PA, United States; ^6^Department of Outpatient Expert Consultation, Pham Ngoc Thach University of Medicine, Ho Chi Minh City, Vietnam

**Keywords:** asthmatic children, asthma control, respiratory polygraphy, OSA, ADHD, AHI

## Abstract

**Background:**

Asthma and obstructive sleep apnea (OSA) are common chronic respiratory disorders in children. The relationship between asthma and OSA is bidirectional; these conditions share multiple epidemiological risk factors. Untreated OSA may cause attention deficit hyperactivity disorder (ADHD) symptoms. This study aimed to assess the prevalence of ADHD in asthmatic children with OSA and the link between asthma control and lung function of children with asthma and OSA.

**Methods:**

A total of 96 children aged 6–15 years diagnosed with asthma, according to the Global Initiative for Asthma (GINA) 2020, were enrolled in this study. All demographic data, including age, gender, body mass index, asthma control status, therapy, the Vanderbilt ADHD Diagnostic Parent Rating Scale, lung function, and exhaled nitric oxide, were collected. In addition, home respiratory polygraphy was used to identify OSA in study subjects.

**Results:**

A total of 96 patients (8.4 ± 2.4 years) were included in the present study. OSA was identified in 60.4% of asthmatic children with a mean apnea-hypopnea index (AHI) of 3.5 ± 3.0 event/h. The inattentive ADHD subtype was significantly lower in the non-OSA asthmatic group than in the OSA asthmatic group (7.9 vs. 34.5%, *p* < 0.05). ADHD had a higher probability of presence (OR: 3.355; 95% CI: 1.271–8.859; *p* < 0.05) in the OSA group (AHI >1 event/h). Children with poorly controlled asthma had a significantly high risk of OSA (83.0 vs. 17.0%, *p* < 0.001) than children with well-controlled asthma. Allergic rhinitis increased the odds of having OSA in patients with asthma [OR: 8.217 (95% CI: 3.216–20.996); *p* < 0.05].

**Conclusion:**

The prevalence of OSA is increased among poorly controlled asthma. ADHD may have a higher prevalence in children with OSA. Therefore, prompt diagnosis of OSA will lead to an accurate asthma control strategy in patients with asthma.

## Introduction

Asthma is one most common major non-communicable diseases in children (1). The prevalence of current wheeze was still high in children and adolescents in low-income countries and increased in lower-middle-income countries (1). Despite advances in asthma control, the asthma-related mortality rate remains high in lower-middle-income countries (2). There is still much work to be done to improve patient education, approach diagnostic tools, and personalize asthma management.

Obstructive sleep apnea (OSA) is defined as repetitive episodes of complete or partial upper airway obstruction during sleep (3). OSA occurs in 1–3% of children (4), especially in the early school and pre-school age, with a peak at the age of 2–8 years and declines in frequency of age (3, 4). The gold-standard diagnostic method for OSA is overnight polysomnography (PSG) or respiratory polygraphy (RPG), with an apnea-hypopnea index (AHI) ≥ 1 event/h associated with the presence of signs and symptoms of OSA (3). Risk factors of childhood OSA are hypertrophy of tonsils, adenoids, and obesity (3, 4).

For the last few decades, several studies have been carried out on the interaction between OSA and other lower airway diseases, especially asthma, in terms of prevalence, pathophysiology, and treatment (5–8). A randomized sample survey of 1,234 children aged 6–14 years in Belgium revealed a 2.0-fold increase in OSA symptoms among children with wheezing (4, 6). However, these two diseases' pathophysiology is likely to overlap because they are all affected by inflammation, neurologic factor, and morphologic manifestation such as obesity (7, 9). Snoring and noisy breathing have been considered the characteristics of OSA in children, but they are also common complaints in asthmatic children (9, 10). On the other hand, airway inflammation and resistance in nocturnal asthma reduced airway flows while sleeping, causing interrupted sleep and/or poor quality of sleep (11). Inversely, systemic corticosteroids for unstable asthma treatment may increase the risk of OSA in children who had both conditions (9). In contrast, OSA plays a role as a contributing mechanism to worsen asthma (11). Ramagopal et al. pointed out that the AHI score was significantly higher than the control in African-American children with poorly controlled asthmatic (12).

In children, OSA might cause intermittent nocturnal hypoxia due to apnea and hypopnea episodes (3), induce cardiovascular disease and metabolic syndrome (3), and lead to increased morbidities and physical development in children with OSA (3, 13). Moreover, Beebe and Gozal suggested that OSA-induced hypoxia and sleep disturbances negatively impact the recovery benefits of sleep (14), cause cellular and chemical imbalance leading to prefrontal cortical dysfunction, and increase neurobehavioral disorders, which can express as overactivity and impulsivity in children (14). Preliminary evidence also suggests that OSA may influence or contribute to attention deficit and hyperactivity disorder (ADHD) symptoms in untreated patients (10, 13). Some research had shown that ADHD and OSA have overlap in diagnosis (13, 15), for example, attention deficit was reported in 95% of pediatric patients with OSA (13), while other research showed a significant link between ADHD and childhood asthma (15). ADHD, OSA, and asthma had a complex relationship, with each syndrome influenced the symptoms of the others (13–15).

This study aimed to examine the clinical characteristics and lung function of asthmatic children with OSA, the prevalence of ADHD among these patients, and the relationship between ADHD and OSA in children with asthma.

## Methods

### Subjects

#### Sample size calculation

Based on the study by Nguyen-Hoang et al. (16), which showed the prevalence of OSA in asthmatic children was 65.9%, the estimated cohort size was done using the following equation:


$$\begin{array}{l} n=Z^{2}\left(1-\alpha /2\right)\times \frac{p\left(1-p\right)}{\Delta^{2}} \end{array}$$

(n = number of subjects; p = expected proportion = 0.65; α = type I error = 0.05; two-sided 95% Confidence Interval, Z = 1.96).

Δ = distance from proportion to limit = 0.1.


$$\begin{array}{l} n=1.96^{2}\times \frac{0.65\left(1-\;0.65\right)}{0.1^{2}}=\;87 \end{array}$$

Approximately 10% of the sample size was added for the possible drop-off and attrition during the study; the sample size was 96.

The convenient sample of 96 children aged 6–15 years visited the Asthma Outpatient Unit of the Immunology-Allergy-Rheumatology department in the National Children's Hospital, Vietnam, for asthma diagnosis and follow-up from 1 August 2020 to 30 June 2021 and were enrolled in this study. Since family medicine is not common in Vietnam, parents could make an appointment directly in the asthma clinic. Asthmatic outpatients had been examined by physicians from the Immunology-Allergy-Rheumatology department. The Global Initiative for Asthma (GINA) guideline for children aged above 5 years had been used for the diagnosis and management of asthma in this study (17).

##### Inclusion criteria

Children aged between 6 and 15 years who were diagnosed with asthma according to the Global Initiatives for Asthma (GINA) 2020 for children aged above 5 years (17) were included in the study.

##### Exclusion criteria

The exclusion criteria were as follows: patient diagnosed with other significant chronic or acute diseases, patient with facial structure malformation, or patient with mental disorders, which caused subjects to be unable to perform spirometry and respiratory polygraphy.

### Methods

#### Study design

**Figure 1:**
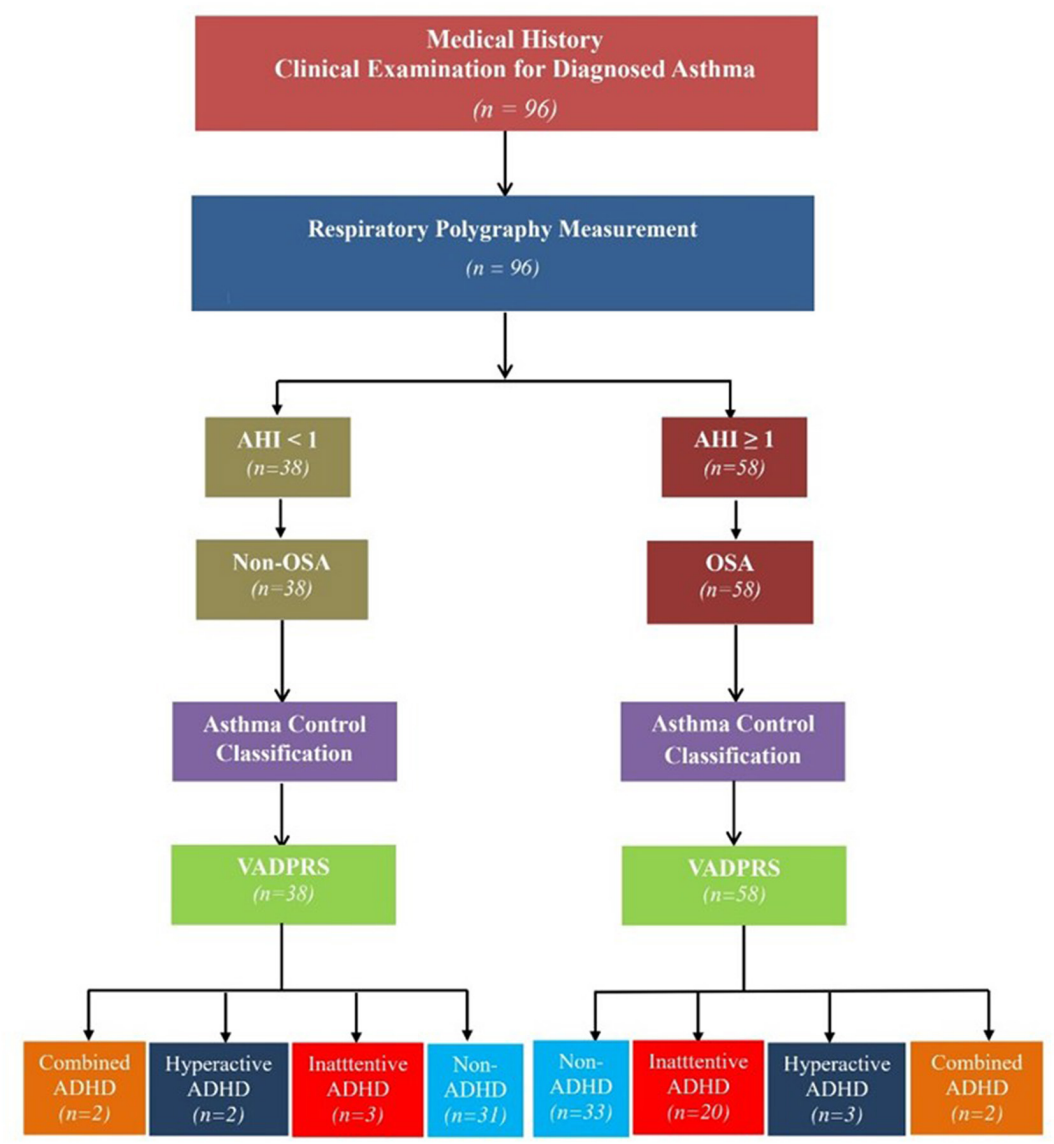
Flowchart of the study procedure. ADHD, attention deficit and hyperactivity disorder; AHI, apnea-hypopnea index; OSA, obstructive sleep apnea; VADPRS, Vanderbilt's ADHD Diagnostic Parent Rating Scale.

It was a cross-sectional and prospective study. The algorithm of the study had been provided in Figure 1.

#### Asthma control selection criteria

The GINA asthma control assessment consists of four questions about asthma symptoms and therapy in the past 4 weeks including frequent use of rescue medications, shortness of breath, nocturnal awakening, and daily activity limitations (17). Based on these symptoms, GINA defined asthma as “well-controlled,” “partly controlled,” or “uncontrolled” asthma. A “poorly controlled” asthma group included “partly controlled” and “uncontrolled” asthmatic children.

The present study used the Asthma Control Test (ACT) for children 12 years and older to measure the level of asthma control (18). The ACT questionnaire evaluates asthma symptoms in the previous 4 weeks. ACT score ≥20 characterizes “controlled asthma,” an ACT score from 16 to 19 indicates “partly controlled asthma,” and an ACT score ≤ 15 indicates “poorly controlled asthma” (18). In the present study, patients with an ACT score < 20 were considered “poorly controlled asthma.” The Vietnamese version of ACT was validated in 2012 (19).

For children older than 4 and younger than 12 years, this study used the c-ACT Test (20). The c-ACT is combined with two parts; the first part has four components and was answered by the child (20). The range of the answers from the first part varied from 0 to 3. The parent or guardian answered the second part with three other components ranging from 0 to 5 (20). The total score of the c-ACT was the sum of all responses, ranging from the value 0 to 27 (20). The value of < 20 was demonstrated as “uncontrolled asthma” (20).

The present study also included information about asthma treatment according to GINA guidelines (17). The medications used by patients with asthma in the present study: inhaled corticosteroids (ICS), ICS-LABA (long-acting beta_2_-agonist), and leukotriene receptor antagonists (LTRA).

#### Anthropometry

Children's weight was measured with a calibrated scale to the nearest 0.1 kg, and height was measured with a stadiometer to 0.1 cm (Medisol, Vietnam). The body mass index (BMI) was calculated, and the BMI z-score was computed using Baylor college of medicine Age-Based Pediatric Growth Reference Charts (https://www.bcm.edu/bodycomplab/BMIapp/BMI-calculator-kids.html) (21). The patients were classified as underweight/normal weight with z-scores between −2 and +0.99, overweight from 1 to 1.99, obese from 2 to 2.99, and very obese ≥3 (22).

#### Lung function testing

The lung function testing (spirometry) was carried out by using Jaeger Vyntus^TM^ IOS (CareFusion, Germany). The spirometry provided the values of forced expiratory volume in 1 s (FEV_1_), forced vital capacity (FVC), and the ratio of FEV_1_ to FVC, which were adjusted according to sex, age, and ethnicity (17). According to the GINA guidelines, the FEV_1_/FVC ratio cut-off of normality was 90% in children (17).

The reversibility of forced expiratory volume in 1 s (FEV_1_) was evaluated after using 200 μg of salbutamol for 15 min. The test was positive when there was an increase in FEV_1_ ≥12% and >200 ml (17).

#### Measuring exhaled NO

The fractional exhaled nitric oxide (FENO) level was measured using Hypair FeNO+ Device (Medisoft; Sorinnes, Belgium) with expiratory airflow of 50, 100, 150, and 350 ml/s. FENO concentrations were classified following the American Thoracic Society/European Respiratory Society (ATS/ERS) recommendation for children: <20 ppb: normal; 20–35 ppb: increased; and >35 ppb: highly enriched (23).

#### Home respiratory polygraphy

The home respiratory polygraphy system used in the study is the Apnea Link^TM^ Plus (Resmed^®^, Australia). The ApneaLink^TM^ Plus could record nasal airflow, snoring, respiratory effort, blood oxygen saturation, and heart rate by nasal cannula, pulse oximetry, and thoracic chest belt.

Parents will be provided careful instruction on how to operate the device and to monitor the child; they had also required to perform several trials before using it at home by themselves. When the device had been returned on the following day, trained physicians who were members of the study board would transfer the raw data files to a computer and score automatically by Apnea Link plus application. A home respiratory polygraphy recording will be deemed valid if the recording duration is ≥ 5 h. Sections with artifacts or poor signals will be excluded from the analysis. If a home respiratory polygraphy is not valid, it will be repeated within the next 7 days.

#### OSA criteria

For all children < 18 years of age, the American Academy of Sleep Medicine defined pediatric OSA with polygraphy by using the apnea-hypopnea index (AHI) ≥ 1 event/h (24). The severity of OSA was classified as recommending: mild OSA: 1 event/h < AHI ≤ 5 event/h; moderate OSA: 5 event/h < AHI ≤ 10 event/h; severe OSA: AHI > 10 event/h (3, 24).

#### Parent-reported ADHD measure

Vanderbilt's ADHD Diagnostic Parent Rating Scale (VADPRS) was chosen to measure behavioral problems in the present study (25). The VADPRS is a parent-reported scale including 55 questions, including all 18 of the DSM-IV criteria for ADHD (25). Each question is put a value on a 4-point scale that describes the frequency of each ADHD symptom (0 = never, 1 = occasionally, 2 = often, and 3 = very often) (25). Besides, the VADPRS includes oppositional defiant disorder (8 items), conduct disorder (14 items), and anxiety/depression (7 items) screening scales (25). Finally, the VADPRS includes performance items that assess functional impairment rated on a 5-point scale (1 = excellent performance and 5 = problematic performance) across academic and social domains (25).

The three subtypes of ADHD based on the score include:

- Predominately Inattentive Subtype: If a child has six or more “Often” or “Very Often” on items from 1 to 9 and less than six for items 10–18, combined with a performance problem (scores of 1 or 2) on questions 48–55.
- Predominately Hyperactive/Impulsive Subtype: If a child has six or more “Often” or “Very Often” on items 10 through 18 and less than six for items 1–9, plus a performance problem (scores of 1 or 2) on questions 48–55.
- Combined Subtype: If a child meets the criteria for both inattentive and hyperactive/impulsive subtypes.

Previous studies have suggested a moderately strong sensitivity of 80% and specificity of 75% for the VADPRS-detecting ADHD in children (26).

#### Data collection

All data of the study subjects including age, gender, BMI score, allergy history, family history, clinical characteristics, measures of spirometry, exhaled NO, therapy, Vanderbilt's ADHD Diagnostic Parent Rating Scale, and respiratory polygraphy parameters were collected and analyzed statistically.

#### Ethical approval

The study was approved by the Hanoi Medical University Institutional Ethical Review Board (502/GCN-HDDDNCYSH-DHYHN) and followed the 1964 Declaration of Helsinki and its later amendments. Informed consent was required from all participants in the study.

#### Statistical analysis

IBM SPSS Statistic 20 software (IBM Corporation, Armonk, NY, USA) has been used to calculate and analyze the collected data. Qualitative data were presented as percentages and analyzed with the chi-squared test. Continuous variables were shown as mean ± standard deviation (SD) and compared with *t*-test between 2 groups and a 1-way analysis of variance among groups, followed by paired comparison with the least-significant difference test. Univariate analysis of associated factors for high-risk OSA in asthma children was performed. All variables with *P* < 0.25 on univariate analysis were included in the multivariate analysis. Odds ratios (ORs) and 95% confidence intervals (CIs) were calculated using logistic regression. A value of *p* < 0 0.05 was considered statistically significant.

## Results

### Descriptive analysis of clinical features of asthma and OSA

During the study period, 96 children with asthma met the inclusion criteria and were enrolled in this study. Their demographic characteristic, comorbidity disease, therapy, and lung function test are shown in Table 1. The mean age was 8.4 ± 2.4 years (6–15 years), including 60.4% of male and 39.6% of female children. Only 10.4% of a subject were overweight or obese (BMI z-scores >1). Approximately 62.5% of the study subjects had a history of allergic rhinitis. Respiratory polygraphy revealed the presence of OSA (AHI> 1 event/h) in 60.4% of study subjects (58 patients) (Table 1). In the present study, 32 patients reported the symptoms of ADHD (33.3%; Table 1).

**Table 1:** Clinical and functional characteristics of study subjects.

**Characteristics (*n* = 96)**	**Mean**
Age (years)	8.4 ± 2.4
Gender (% male)	58 (60.4%)
BMI (kg/m^2^)	17.1 ± 2.2
BMI z-scores >1, *n* (%)	10 (10.4)
ACT score	20.7 ± 3.7
**Asthma control**, ***n*** **(%)**	
Well-controlled	49 (51.0)
Partly or uncontrolled	47 (49.0)
**Therapy**, ***n*** **(%)**	
ICS, *n* (%)	67 (69.8)
ICS-LABA, *n* (%)	15 (15.6)
Montelukast, *n* (%)	72 (75.0)
Allergic rhinitis, *n* (%)	60 (62.5)
ADHD, *n* (%)	32 (33.3)
FEV1 (%pred)	86.1 ± 16.1
FVC (% pred)	92.6 ± 15.1
FEV1/FVC (%)	93.1 ± 11.5
PEF (% pred)	69.1 ± 16.2
Bronchial FENO (ppb)	21.0 ± 12.9
**Respiratory polygraphy**	
AHI ≤ 1/h, *n* (%)	38 (39.6)
1 < AHI ≤ 4/h, *n* (%)	35 (36.5)
5 < AHI ≤ 9/h, *n* (%)	15 (15.6)
AHI ≥ 10/h, *n* (%)	8 (8.3)

ACT, asthma control test; AHI, apnea-hypopnea index; BMI, body mass index; FENO, fractional exhaled nitric oxide; FEV_1_, forced expiratory flow in 1 s; FVC, forced vital capacity; PEF, peak expiratory flow; ppb, part per billion.

Among 58 asthmatic children with OSA (60.4%), 36.5% were mild OSA (AHI= 1–4 events/h), 15.6% were moderate OSA (AHI = 5–9 events/h), and 8.3% were classified as severe OSA (AHI ≥ 10 event/h). The average AHI index was 3.45 ± 3.01 event/h (Table 2).

**Table 2:** Demographic characteristics and asthma control levels of the study subjects classified by OSA.

**Characteristics**	**OSA (+)**	**OSA (-)**	***p*** **-Value**
*n*	58	38	
Age (years)	8.5 ± 1.5	8.3 ± 2.2	0.478
Male (female), ratio	38/20 (1.9)	20/18 (1.1)	0.286
Allergic rhinitis (*n*, %)	47 (81.0)	13 (34.2)	0.001
BMI score	17.2 ± 3.0	17.4 ± 2.9	0.735
BMI z-scores >1(*n*, %)	7 (12.1)	3 (7.9)	0.614
ADHD (*n*, %)	25 (43.1)	7 (18.5)	0.015
FEV1 (%pred)	75.2 ±16.6	82.6 ± 15.1	0.544
FEV1/FVC (%pred)	64.7 ± 19.0	68.7 ± 11.8	0.537
PEF (%pred)	62.2 ± 11.4	69.2 ± 16.3	0.812
Bronchial FENO, ppb	21.6 ± 12.0	14.2 ± 11.6	0.03
**Asthma (** ***n*** **, %)**			
Well-controlled	19 (38.8)	30 (61.2)	
Partly or uncontrolled	39 (83.0)	08 (17.0)	0.001
**Therapy (** ***n*** **, %)**			
ICS (*n*, %)	40 (69.0)	27 (71.0)	0.695
ICS-LABA (*n*, %)	10 (17.2)	5 (13.2)	0.842
Montelukast (*n*, %)	45 (77.6)	27 (71.1)	0.365
ACT-score	18.2 ± 3.2	23.8 ± 3.5	0.007
**Respiratory polygraphy**			
AHI (mean)	3.5 ± 3.0	0.5 ± 0.2	
Lowest oxygen saturation, %	82.7 ± 24.1	84.3 ± 28.2	
1 < AHI ≤ 5/h, *n* (%)	35 (36.5	-	
5<AHI ≤ 10/h, *n* (%)	15 (15.6)	-	
AHI > 10/h, *n* (%)	8 (8.3)	-	

ACT, asthma control test; AHI, apnea-hypopnea index; BMI, body mass index; FENO, fractional exhaled nitric oxide; FEV_1_, forced expiratory flow in 1 s; FVC, forced vital capacity; PEF, peak expiratory flow; ppb, part per billion.

### Prevalence of OSA as comorbidity, relationship with asthma control, and other risk factors

The differences between asthmatic children with OSA and those without OSA are shown in Table 2. Children with respiratory polygraphy evidence of OSA were overall anthropometrically similar to those without OSA. However, the frequency of reported symptoms of allergic rhinitis in the asthmatic children with the OSA group was significantly higher than those without OSA (81.0 vs. 34.2%; *p* < 0.05) (Table 2).

A significantly higher prevalence of OSA was found among the poorly controlled asthma group than the well-controlled asthma group (83.0 vs. 17.0%, *p* < 0.001). In addition, the mean value of ACT score in non-OSA was statistically significantly lower than the OSA group (18.2 ± 3.2 vs. 23.8 ± 3.5, *p* < 0.05), reporting a correlation between asthma control and OSA.

#### Clinical and functional characteristics of asthmatic children with OSA classified by asthma control status

Among those who had OSA, age was associated with a well-controlled asthma status (9.22 ± 2.31 vs. 7.28 ± 3.15, p < 0.05) (Table 3). In addition, children with well-controlled asthma had lower bronchial FENO (18.65 ± 16.75 vs. 33.24 ± 13.21, *p* < 0.05) and higher ACT scores (24.89 ± 5.47 vs. 19.89 ± 4.34, *p* < 0.05) than children with poorly controlled asthma (Table 3). Children with well-controlled asthma also had lower AHI scores than the children with poorly controlled asthma (1.79 ± 1.01 vs. 4.85 ± 1.87, *p* < 0.05).

**Table 3:** Characteristics of the asthmatic group with OSA classified by asthma control status.

**Characteristics**	**Well-controlled asthma**	**Partly or uncontrolled asthma (-)**	***p*** **-Value**
N	23	35	
Age (years)	9.22 ± 2.31	7.28 ± 3.15	0.012
Male (female), ratio	14/9 (1.6)	24/11 (2.2)	0.409
Allergic rhinitis (*N*, %)	17 (73.91)	30 (85.71)	0.532
BMI score	16.92 ± 4.87	17.34 ± 3.95	0.127
Obesity (*N*, %)	4 (17.39%)	3 (8.57)	0.544
FEV1 (%pred)	81.51 ± 19.18	77.56 ± 20.41	0.337
FEV1/FVC (%pred)	85.48 ± 21.55	80.55 ± 19.0	0.134
PEF (%pred)	77.23 ± 22.14	72.57 ± 25.67	0.708
Bronchial FENO, ppb	18.65 ± 16.75	33.24 ± 13.21	0.0075
ACT-score	19.89 ± 4.34	24.89 ± 5.47	0.01
AHI score	1.79 ± 1.01	4.85 ± 1.87	0.022

ACT, asthma control test; AHI, apnea-hypopnea index; BMI, body mass index; FENO, fractional exhaled nitric oxide; FEV_1_, forced expiratory flow in 1 s; FVC, forced vital capacity; PEF, peak expiratory flow.

#### Odds ratio of OSA for the study subjects

**Figure 2:**
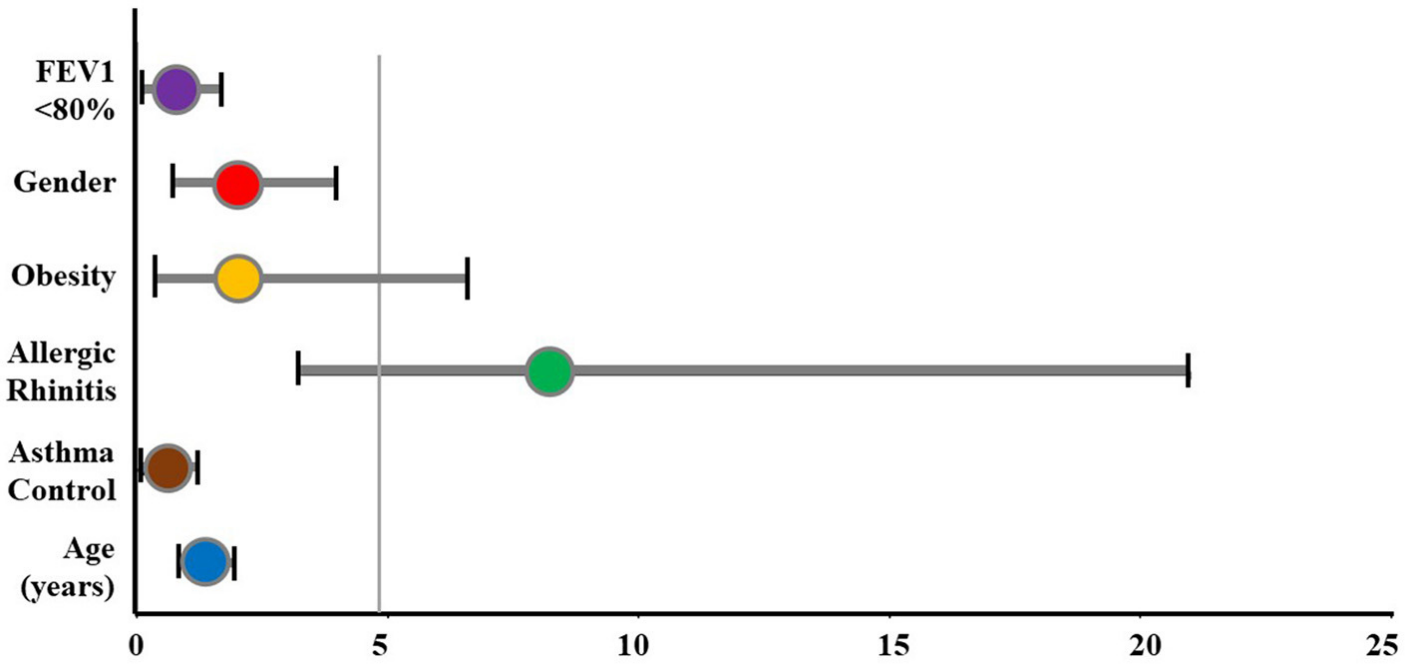
The odds ratio of OSA for study subjects. FEV1, forced expiratory volume in 1 s; OSA, obstructive sleep apnea.

The OR of OSA in children with asthma was evaluated using multivariate logistic regression (Table 4 and Figure 2). The prevalence of OSA increased in the group of asthmatic children with allergic rhinitis (OR: 8.217; 95% CI: 3.216–20.996; *p* < 0.05). Gender, obesity, FEV_1_, and asthma control were not associated with the risk of OSA in subjects with asthma.

**Table 4:** Odds ratio of OSA for asthmatic children.

**Characteristics**	**OR**	**95% CI**	***p*** **-Value**
Age (years)	1.025	0.799–1.315	0.845
Asthma controlled	0.130	0.050–0.337	0.097
Allergic rhinitis	8.217	3.216–20.996	0.000
Obesity	1.601	0.387–6.620	0.735
Gender	1.710	0.741–3.945	0.286
FEV_1_ < 80%	0.383	0.165–0.888	0.06

FEV_1_, forced expiratory flow in 1 s; OR, odds ratio.

### Prevalence of ADHD and association with OSA comorbidity

**Figure 3:**
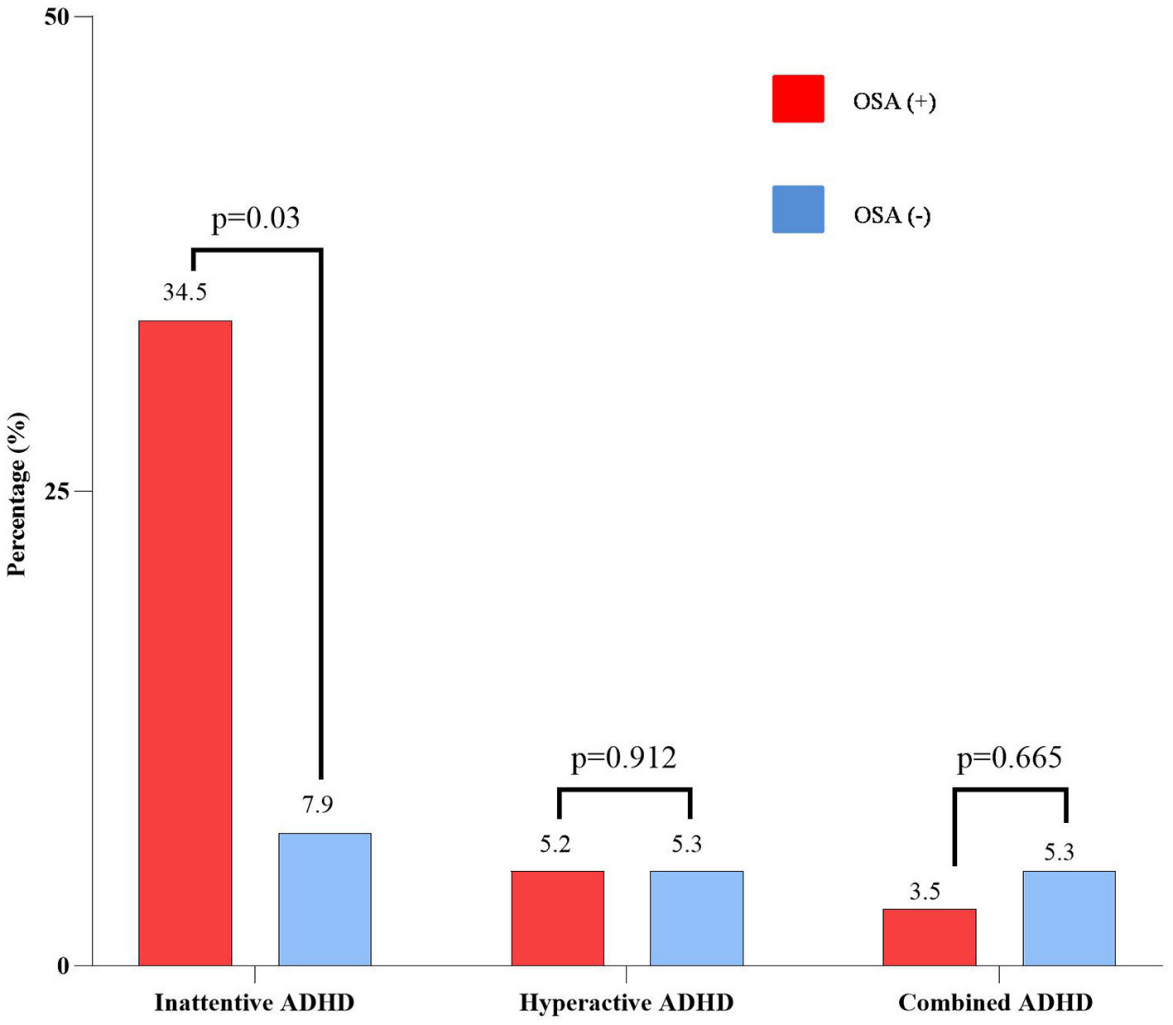
Parent-reported ADHD symptoms of the study subjects classified by OSA. ADHD, attention deficit and hyperactivity disorder; OSA, obstructive sleep apnea.

Parent-reported symptoms of ADHD by the VADPRS are given in Figure 3. The frequency of parent-reported symptoms of ADHD in asthmatic children with OSA was significantly higher than in those without OSA (43.2 vs. 18.5%, *p* < 0.05). Among asthmatic patients with OSA, 34.5% had a symptom of Inattentive ADHD, which was significantly higher than the non-OSA group (34.5 vs. 7.9%, *p* < 0.05). There were no significant differences between the OSA and non-OSA groups for the prevalence of hyperactive/impulsive ADHD or other behavioral problems.

There were no noticeable differences in age, gender, BMI score, allergic rhinitis, and asthma control between the OSA + ADHD and OSA without ADHD groups (Table 5). However, OSA + ADHD group had significantly higher AHI scores (*p* < 0.05) and marked lower SaO_2_ (*p* < 0.05) as compared with the OSA group.

**Table 5:** Demographic characteristics, asthma control, and respiratory polygraphy of the study subjects classified by parent-reported ADHD symptoms.

**Characteristics**	**OSA + ADHD**	**OSA alone**	***p*** **-Value**
*n*	25	33	
Age (years)	8.9 ± 1.2	7.9 ± 3.4	0.380
Male (female), ratio	17/8 (2.1)	21/12 (1.8)	0.665
Allergic rhinitis (*n*, %)	21 (84.0)	26 (78.8)	0.112
BMI score	17.6 ± 3.3	17.1± 3.5	0.225
**Asthma (** ***n*** **, %)**			
Well-controlled	10 (43.5)	13 (56.5)	
Partly or uncontrolled	15 (42.9)	20 (57.1)	0.174
ACT-score	18.9 ± 4.7	19.9 ± 5.3	0.549
**Respiratory polygraphy**			
AHI (mean)	4.1 ± 2.6	2.2 ± 1.8	0.042
Lowest oxygen saturation, %	68.6 ± 29.5	84.6 ± 34.1	0.0063

ADHD, attention deficit and hyperactivity disorder; AHI, apnea-hypopnea index; BMI, body mass index; OSA, obstructive sleep apnea.

#### Odds ratio of ADHD for the study subjects

**Figure 4:**
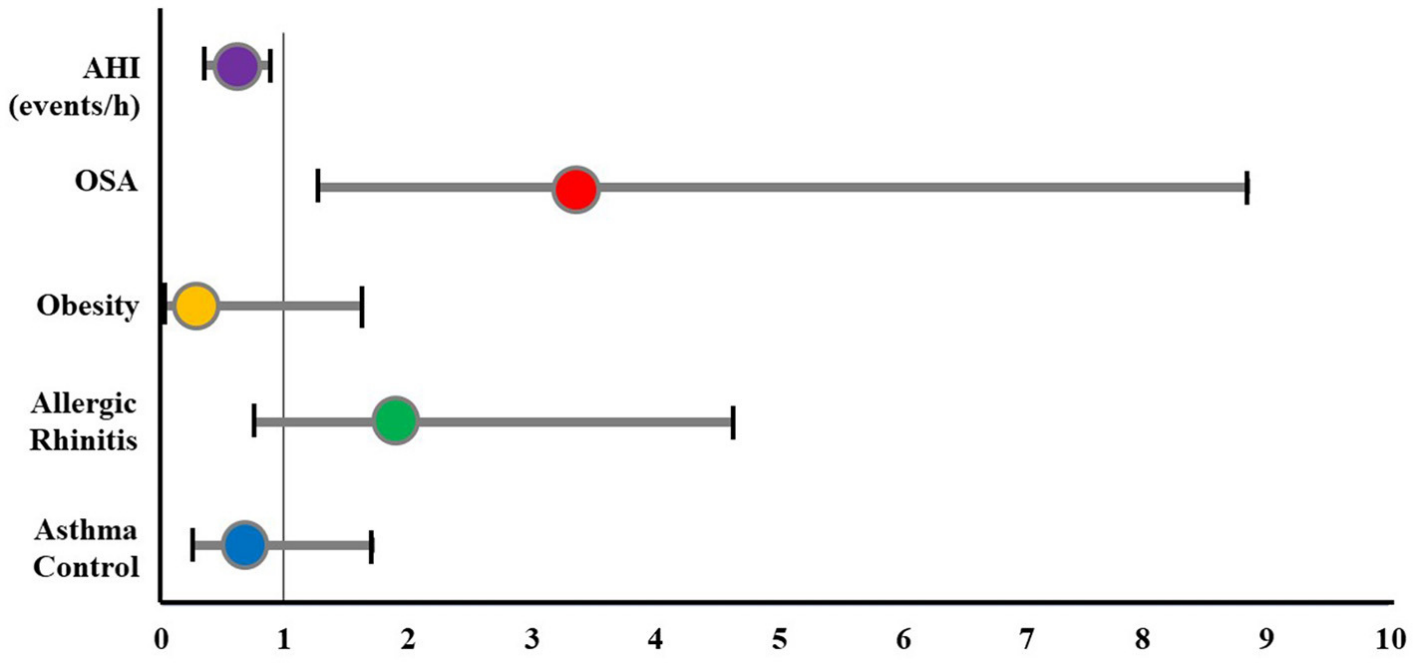
The odds ratio of ADHD for study subjects. ADHD, attention deficit and hyperactivity disorder; AHI, apnea-hypopnea index; OSA, obstructive sleep apnea.

The OR for ADHD in study subjects was analyzed using logistic regression (Table 6 and Figure 4). The result showed that the prevalence of ADHD increased in the subject with OSA (OR: 3.355; 95% CI: 1.271–8.859; *p* < 0.05). However, asthma control, allergic rhinitis, and obesity were not associated with the prevalence of ADHD.

**Table 6:** Odds ratio of ADHD for study subjects.

**Characteristics**	**OR**	**95% CI**	***p*** **-Value**
Asthma controlled	0.671	0.261–1.724	0.477
Allergic rhinitis	1.865	0.746–4.663	0.263
Obesity	0.197	0.024–1.630	0.157
OSA (AHI ≥1 event/hour)	3.355	1.271–8.859	0.015
AHI (event/hour)	0.606	0.503–0.731	0.000

ADHD, attention deficit and hyperactivity disorder; AHI, apnea-hypopnea index; OR, odds ratio; OSA, obstructive sleep apnea.

## Discussion

This present study emphasizes that asthmatic patients had a high prevalence of OSA as compared with the maximal estimated prevalence of OSA in a non-asthmatic population (60.4 vs. 4.0%, *p* < 0.01) (3, 4). The diagnosis of OSA in this study is based on AHI measurement following the American Academy of Sleep Medicine guideline (3, 24). The mean AHI of study subjects measured by respiratory polygraphy is 3.5 ± 3.0 events/hour and is considered mild OSA. Several studies have shown that OSA is common in the asthmatic population (3, 5, 6, 15). In one study, Kheirandish-Gozal et al., subjected 92 poorly controlled asthma children between 3 and 10 years to overnight polysomnography (2). OSA was present in 58 patients, with a prevalence of 63% (3). In another study, the majority of OSA in Vietnamese asthmatic children is 65.9% (16).

Many studies have reported the bidirectional connection between OSA and asthma, including incidence, risk factors, pathophysiology, and treatment (5, 7, 8). In the present study, we found that the coexistence of OSA (diagnosed based on respiratory polygraphy) was significantly more frequent in poorly controlled asthma children than in the well-controlled asthma group (83.0 vs. 17.0%, *p* < 0.001). It means that poorly controlled asthma was able to be a risk factor for OSA in children. These findings are similar to other previous studies (5, 27). In a recent study of 203 children with asthma aged 3–16 years (27), there was a significantly higher prevalence of OSA in the poorly controlled asthma group than in children with well-controlled asthma (34.2 vs. 13.97%, *p* < 0.01) (27). Another large multicentric cross-sectional study was conducted to investigate the frequency of asthma and sleep-disordered breathing among school-aged children in China (5). The authors demonstrated that sleep-disordered breathing, such as chronic snoring (OR = 1.28, 95%CI: 1.01–1.62), and OSA (OR = 1.92, 95%CI: 1.34–2.76) were significantly associated with asthma, after adjusting for potential confounding factors (5).

Studies in children with OSA have investigated the association of OSA with systemic inflammation and localized inflammation of the upper airway tissue (28, 29). Systemic inflammation has been associated with OSA by studies that have identified upregulation of plasma CRP, increased neutrophils in the sputum, increased urinary levels of cysteinyl leukotriene, and increased levels of leukotrienes and prostaglandins in exhaled breath condensate in children with OSA (29). At present, asthma had been proven to be an inflammatory disease (30). The higher prevalence of OSA in children with asthma indicates that this high-frequency co-infection requires special attention because it can make asthma more difficult to control (4, 17). In asthmatic patients, OSA plays a role as a contributing factor to aggravating asthma because airway obstruction in nocturnal asthma is linked to disturbances in sleep distribution, difficulty sleeping, insomnia, early waking up, and daytime sleepiness (9–11). However, there are still many arguments about the bidirectional interaction between asthma and OSA (9).

Obesity is considered an independent risk factor for both asthma and OSA; moreover, obesity makes a strong link between asthma and OSA (7–9). In the present study, only a few children with overweight or obese in the asthma group with OSA (Table 2). This result was different from other studies in Western countries, which showed that high BMI increased the prevalence of OSA (3, 4, 13), but similar to other Vietnamese studies (16, 31).

Numerous studies demonstrate that allergic rhinitis and asthma usually coexist (1, 2, 17). In the present study, 62.5% of the comorbidities were allergic rhinitis. The frequency of reported symptoms of allergic rhinitis in the asthmatic children with the OSA group was significantly higher than those without OSA (81.0 vs. 34.2%; *p* < 0.05). Previous studies in sleep-related problems had established the role of nasal obstruction due to allergic rhinitis in increasing the risk of upper airway obstruction during sleep, snoring, and OSA (32). The results of measuring the OR in the present study demonstrated that allergic rhinitis was the significant risk for the presence of OSA (OR: 8.217; 95% CI: 3.216–20.996; *p* < 0.05) (Figure 2).

The GINA guidelines emphasized the role of the lung function test to determine the level of asthma control (17); however, the relationship between asthmatic's patient lung function results and OSA is unclear (32). In a previous study (33), Sheen et al. enrolled 220 asthmatic children and revealed that FEV1/FVC is associated with the pediatric sleep questionnaire score (33). However, another research did not find any difference between the lung functions of an asthmatic patient with a high risk of OSA and those with a low risk of OSA (32). In the present study, we got the same result (Table 2). This also suggests that the degree of obstruction of the lower airway (bronchus) is not related to the severity of OSA in asthmatic children with OSA. Therefore, spirometry alone does not screen or suspect patients with OSA or the severity of OSA. The pathogenesis of OSA explains this due to the predominant obstruction of the upper respiratory tract (3).

High bronchial FENO in asthmatic children is a marker for allergic inflammation due to eosinophilia that has not been well-controlled by ICS or requires elevating ICS dose (23). However, a systematic search collected studies published from 1996 to 2016 from the PubMed, EMBASE, the Cochrane Library, and MEDLINE databases (34) revealed that FENO levels were significantly higher in patients with OSA compared to that in the control groups (6.32 ppb, 95% CI: 4.46–8.33, *p* < 0.001) (34). Consequently, elevating bronchial FENO would be a biological marker for suspecting OSA in a well-controlled asthmatic patient (34). OSA treatment with long-term CPAP therapy also reduced FENO levels (−5.82 ppb, 95% CI: −9.6 to −2.01, *p* < 0.001) (34). In the present study, the average bronchial expiratory nitrite concentration (FENO) of the asthmatic group with OSA was higher than the asthmatic group without OSA (21.6 ± 12.0 ppb vs. 14.2 ± 11.6 ppb, *p* < 0.05) (Table 2).

Uncontrolled OSA in children may cause adverse physical and mental consequences, especially ADHD or ADHD-like symptoms (13). The prevalence of ADHD is about 3% worldwide, while almost 95% of pediatric OSA patients had attention deficit disorders (13). The present study pointed out a significantly higher prevalence of the inattentive ADHD subtype in the asthmatic group with OSA (Figure 3). OSA was considered as a risk for the presence of ADHD (OR: 3.355; 95% CI: 1.271–8.859; *p* < 0.05), rather than asthma control (Figure 4). This result was similar to other studies (31, 35). Moreover, OSA + ADHD group had a markedly higher AHI score and significantly lower saturation oxygen than the OSA-alone group. Previous studies suggested that hypoxia in OSA is related to ADHD (13, 14). Sleep apnea may increase the rapid movement eye (REM) ratio and decrease the nocturnal SaO_2_ (14), which may cause brain function impairment (14). This dysfunction leads to cognitive failure, executive disorders, and emotional disorders (13, 14), which play an important role in the pathophysiology of ADHD in children with OSA (3, 13, 14). Although hyperactive ADHD is more common in children aged below 6 years, in the present study, the prevalence of children with combined ADHD and hyperactive ADHD were 5.2 and 4.2%, respectively. This could be due to the parents just thinking that their children were naughty. Therefore, when children had poor academic performance, their parents might take them to the hospital for consultation regarding the diagnosis of ADHD. However, further studies with a large number of patients should be done to measure the exact prevalence of ADHD in children with asthma and OSA.

This study, though, was subjected to some limitations. First, the present study was an observational, cross-sectional study with a small sample size. Moreover, overnight polysomnography or respiratory polygraphy is still expensive and time-consuming. Consequently, the diagnosis of pediatric OSA in Vietnam is still difficult and requires a lot of effort. Second, the present study missed gastroesophageal reflux, one of the most common risk factors for both asthma and OSA in children. Finally, because the present study was a cross-sectional study, the role of the patient's therapy in ADHD symptoms of study subjects was investigated. Thus, further studies should be conducted to clarify these limitations.

## Conclusion

In summary, we reported the initial observations that the prevalence of OSA is significantly high in poorly controlled asthmatic children. Allergic rhinitis is also associated with a higher risk of OSA. The bidirectional relationship between asthma and OSA may make it exceedingly difficult to manage, especially in children with asthma–OSA overlap associated with ADHD. Thus, both diseases should be diagnosed and treated promptly.

## Data availability statement

The original contributions presented in the study are included in the article/supplementary material, further inquiries can be directed to the corresponding author.

## Ethics statement

The studies involving human participants were reviewed and approved by Hanoi Medical University Institutional Ethical Review Board (IRB-VN01.001/IRB00003121/FWA00004148). Written informed consent to participate in this study was provided by the participants' legal guardian/next of kin.

## Author contributions

LN-N-Q, MN-T-T, MN-T-P, CL-Q, HL-T-M, and SD-Q: conceptualization, validation, writing the original draft preparation, methodology, writing, reviewing, and editing. LN-N-Q, MN-T-T, and SD-Q: software. LN-N-Q, MN-T-T, MN-T-P, CL-Q, and SD-Q: formal analysis. All authors contributed to the article and approved the submitted version.
